# Computational Studies of the Intestinal Host-Microbiota Interactome

**DOI:** 10.3390/computation3010002

**Published:** 2015-01-14

**Authors:** Scott Christley, Chase Cockrell, Gary An

**Affiliations:** Department of Surgery, University of Chicago, 5841 South Maryland Avenue, Chicago, IL 60637, USA

**Keywords:** translational systems biology, translational bioinformatics, microbiome, mathematical modeling and simulation

## Abstract

A large and growing body of research implicates aberrant immune response and compositional shifts of the intestinal microbiota in the pathogenesis of many intestinal disorders. The molecular and physical interaction between the host and the microbiota, known as the host-microbiota interactome, is one of the key drivers in the pathophysiology of many of these disorders. This host-microbiota interactome is a set of dynamic and complex processes, and needs to be treated as a distinct entity and subject for study. Disentangling this complex web of interactions will require novel approaches, using a combination of data-driven bioinformatics with knowledge-driven computational modeling. This review describes the computational approaches for investigating the host-microbiota interactome, with emphasis on the human intestinal tract and innate immunity, and highlights open challenges and existing gaps in the computation methodology for advancing our knowledge about this important facet of human health.

## 1. Introduction

The Human Microbiome Project (HMP) and Metagenomics of the Human Intestinal Tract (MetaHIT) are two large-scale data collection projects that have helped to spur research on the microbial communities that inhibit various niches of the human body [1-3]. Of the epithelial surfaces where microbes reside, the intestinal microbiota is one of the most diverse communities consisting of hundreds of species, and its composition varies between individuals as well as across space and time within the same individual [2,4-7]. The intestinal microbial community is established during infancy and coevolves with the host immune system into a symbiotic relationship [8-11]. Disturbance of this host-microbiota relationship has been implicated or suggested in numerous diseases such as inflammatory bowel disease [12], necrotizing enterocolitis [13], gut-derived sepsis [14], and cancer [15,16].

**Figure 1.**
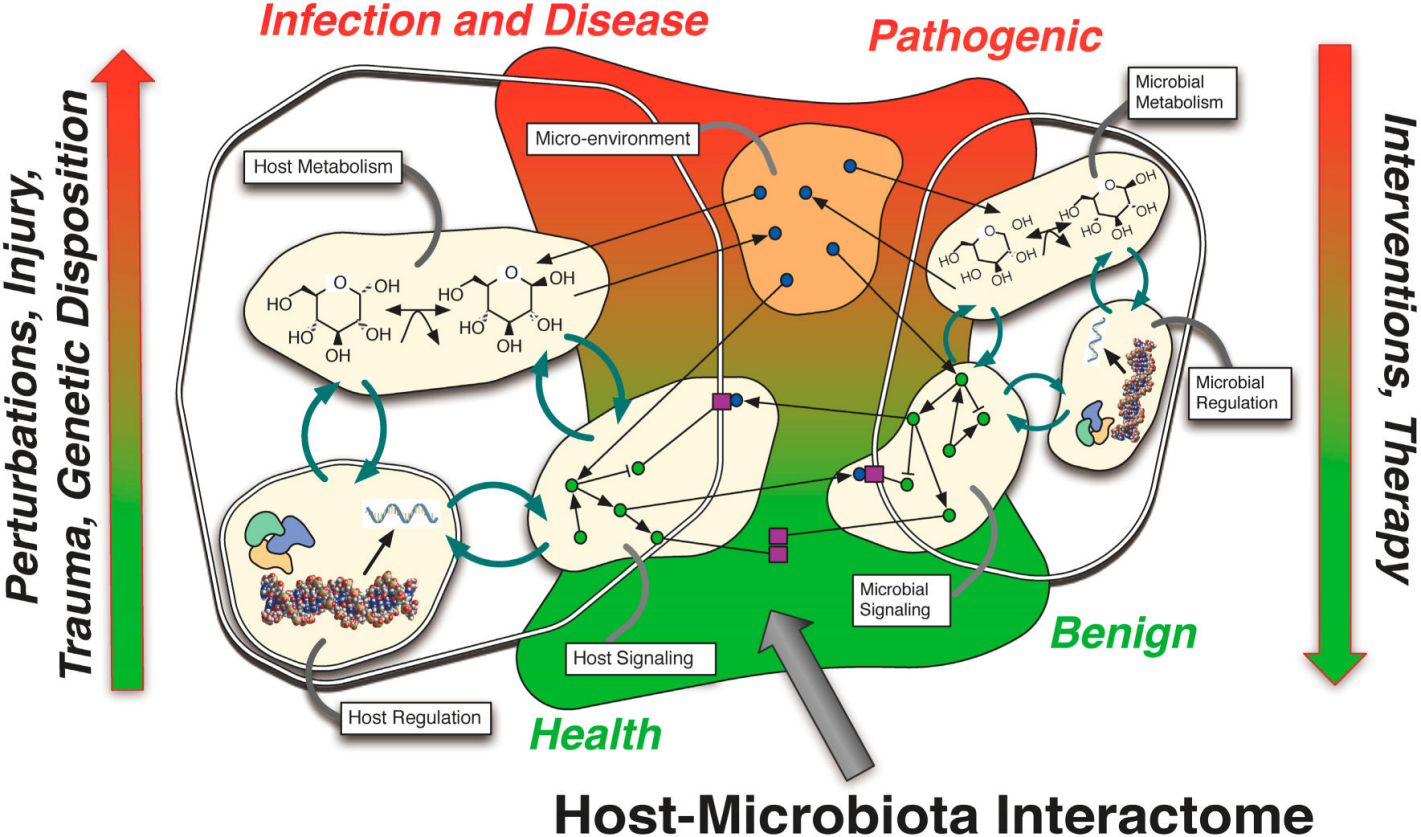
The Host-Microbiota Interactome. The homeostasis of a healthy host and a benign microbiota can shift towards infection and disease in the host and a pathogenic microbiota due to many factors such as injury, trauma and genetic disposition, while interventions and therapy can shift it back. In actuality, there is a spectrum of physiological states, perturbations and interventions between those two extremes as abstractly represented by the color gradient. The host-microbiota interactome consists of multiple inter-connected components. Metabolic, signaling and regulatory components are shown as tightly-coupled processes within host and microbial cells. Extracellular metabolite exchange and competition occurs in the micro-environment shared by host and microbial cells, and these metabolites can directly affect metabolism and signaling processes. Signaling pathways within host and microbial cells secrete molecules into the extracellular environment, sense the local environment by processing extracellular molecules that bind to cell receptors, and mediate physical interactions between cells.

The primary hypothesis of the pathogenesis of these intestinal disorders invokes a series of stages in the progression from a healthy state to disease, whereby external perturbations, genetic predispositions and host-microbiota feedback interactions lead to a self-sustaining chronic state involving host dysfunction and microbiota dysbiosis. We consider a healthy state as one where the host intestinal architecture and immune system are in complementary homeostasis with the commensal microflora. This is a dynamic steady-state as the microbial community composition undergoes changes due to dietary input as well as host nutrient requirements throughout the typical work/sleep circadian rhythm, yet these changes are typically well within the normal robust boundaries of a healthy system. The divergence from a healthy state starts with the introduction of a significant perturbation that disturbs this homeostasis. It could be a host perturbation such as an injury, trauma, surgery or exposure to harsh chemicals, essentially anything that activates a systemic host immune response that reaches the intestinal cells. It could also be a microbiota perturbation due to the ingestion of toxins, tainted or poisoned food, or medicines and other drugs. Small perturbations may become further exacerbated in some individuals due to genetic susceptibility, likewise it may be a combination of perturbations through diet, antibiotic regiments, chemotherapy or radiation treatments that lead to a disruption of the baseline healthy homeostasis. Interactions that feed back and forth between the host and the microbiota, which may involve multiple steps and transitions, eventually either lead back to a healthy homeostasis or head along a disease trajectory. Along the disease trajectory the commensal microbiota may enter a dysbiotic state by shifting towards a pathobiome, which further enforces a pathogenic host response [17]. It is this interactome that is the current “black box” of the intestinal system: peering into and manipulating this black box holds the promise of designing interventions that can control the interaction dynamics and guide the system towards a healthy outcome (Figure 1). For patients with existing chronic conditions, appropriate control interventions would aim to steer patients back towards healthy homeostasis, or at least manage the interactome to prevent the outbreak of severe disturbances and relapses. There are numerous review articles that discuss the underlying biology for each of the different aspects of the host-microbiota interactome including the role of the immune system [18-20], host genetics [21,22], protection from pathogens by the microbiota [23], microbial community interactions [24-26], the microbiota’s influence on development [27] and relationship with pathogenesis [28,29]. Disentangling the complex multi-stage web of interactions that lead to these diseases will require novel approaches, using a combination of data-driven bioinformatics with knowledge-driven computational modeling.

Data-driven bioinformatics refers to the large body of analysis techniques and tools that seek to discover meaningful patterns from biological data. These techniques include a wide-range of statistical, mathematical and algorithmic methods. Bioinformatic tools are considered “data-driven” because of their common feature of using biological data as the primary input for their processing, and the output from these tools provide knowledge to better understand that data. In general, these tools are designed to operate on data produced by various high-throughput technologies, which generate a large quantity of experimental data, but other sources of data such as biological databases and literature are also applicable. The breadth and scope of bioinformatics has led to the numerous “omics” subfields based upon the type of biological data, *i.e.*, genomics for genome data, proteomics for protein data, transcriptomics for gene transcription data, *etc.* Knowledge-driven computational modeling seeks to understand biological systems and their behavior by simulating the cause and effect relationships of biological mechanisms using mathematical methods. “Knowledge-driven” refers to the process of constructing the model that includes a primary task of encoding knowledge about biological entities, processes and mechanisms into mathematical objects, and thus the model becomes an abstract representation of a biological system. The simulation of a model imitates the biological system over time and (possibly) space and allows for the behavior of the system to be analyzed. Specifically, models can be incorporated with hypothetical mechanisms and interventions to evaluate their plausibility and predict their efficacy. Combination of data-driven bioinformatics with knowledge-driven computational modeling will be crucial to understand the host-microbiota interactome because neither methodology is sufficient alone. Bioinformatics is needed to extract insights from experimental data and suggest new hypotheses but is incapable of evaluating the causal outcome of those hypotheses, which computational modeling can provide. Likewise, computational models are abstractions of the real biological system and require experimental data to validate and calibrate their behavior, and they produce simulated data that looks much like experimental data, which requires bioinformatics to properly analyze. This review covers both methodologies in the study of the host-microbiota interactome and highlights efforts to combine the two approaches.

As the generation of high-throughput experimental data has become relatively inexpensive (though there are often sample collection hurdles to be overcome), analysis of that data has quickly become the primary bottleneck to extract meaningful knowledge. Construction of specialized databases such as IMG/M [30], SEED [31] and Greengenes [32] have consolidated annotated genomic data for bacteria, and some provide analytical pipelines (*i.e.*, RAST [33], MG-RAST [34]). However, these tools provide only an initial, first-stage descriptive analysis of the data. Customized scripts and programs are still often required for performing in-depth bioinformatics analysis. In the future, projects like KBase will provide an open development environment based upon a standard data and service model for customized analysis pipelines [35]. Moving further down the chain of extracting knowledge from data, there is even less standardization for dynamic computational modeling, which is aimed at examining the behaviors associated with imputed bio-molecular mechanisms. While the efforts of the Systems Biology Markup Language (SBML) [36] and BioModels repository [37] have helped to standardize the use and simulation of biochemical reaction models, they are too specific to cover the broader range of biological modeling. Describing the various modeling methods and specific models using mathematical notation within the research article remains the primary mode of dissemination, with the implementation of the actual models often left as an exercise for the reader.

In this review, we will focus on the computational approaches for investigating the microbiota and the host-microbiota interactome. Many of the current bioinformatic tools are in their early stages, often providing mostly descriptive analysis, yet there is progress towards increasing functional detail that will eventually lead towards mechanistic predictive models. Our objective is not to describe all of the existing tools and methods in detail, nor the protocols for performing the various analyses, though we will reference existing articles with this information when available. Instead we will highlight the current open challenges in the field and existing gaps in the integration of multiple techniques, with the hope that it will encourage research in new computational methods.

## 2. Microbiota Studies

Following ecology standards, the term “microbiota” is used to refer to the collection of microbial species that comprise a specific ecological niche, while the term “microbiome” refers to the genomic content of those microbiota. Many research papers tend to intermix the two terms, so careful reading of the context is often required to differentiate the appropriate interpretation. Computational studies of the microbiota have primarily been DNA-based either with marker gene (*i.e.*, 16S and 18S) profiling or metagenomics [38,39]. Marker gene profiling, also called amplicon sequencing, uses the known properties of the variable regions within ribosomal RNA (rRNA) genes that are present within all bacteria to provide a taxonomic characterization and relative abundance of the microbial community. Metagenomics performs sequencing of the entire genomic DNA and thus can provide a complete repertoire of genes within the microbiome. DNA-based techniques have been useful in characterizing the composition of the microbiome, especially in comparative studies of treatments and experimental conditions. Metatranscriptomics is an RNA-based approach that has become more practical with the decreasing cost of sequencing. Similar to RNA-seq used for eukaryotic organisms [40,41], the RNA of all bacterial species is captured in an unbiased way without requiring existing genome sequences with annotated genes as with microarray technology. Metatranscriptomics offers the promise of revealing the dynamics of microbial communities, however there are still significant experimental and computational hurdles [42,43].

While the bioinformatic analysis of microbiota is being rapidly adopted by the biological sciences, dynamic computational modeling and simulation has lagged behind. One bright spot is the development of genome-scale metabolic models for model bacteria organisms [44], and the constraint-based optimization method called flux balance analysis for predicting the flow of metabolites and compounds through a network of metabolic reactions [45,46]. These predictive models can reconcile culture conditions and phenotypes to experimental data, and offer intriguing possibilities for enhancing individual species and microbial community discovery [47]. On the other hand, most dynamic computational models have focused on an individual species and a specific signaling pathway or behavior within that species such as quorum-sensing, biofilm formation, motility or colony pattern formation. Recent research has started to tackle the more sophisticated problems of multi-species interactions [48,49] and whole-cell simulations [50].

### 2.1. Marker Gene Profiling

Marker gene profiling (also called gene amplicon sequencing) requires DNA to be extracted from the samples, and large collection projects such as the Earth Microbiome Project recommend a standard experimental protocol [51]. Universal primers specific to the ribosomal RNA gene are then used to amplify the DNA sequence, while barcode sequences are also incorporated thus allowing multiple samples to be multiplexed on a single sequencing run. While initially using 454 pyrosequencing technology, amplicon studies have steadily switched to Illumina sequencing which provides equivalent results and greater coverage [52]. The computation tools have advanced quickly with pipelines such as QIIME [53] and MEGAN [54] providing many standard analytical techniques; even so there is continuing work to provide greater accuracy and fidelity [55,56]. Two primary limitations are: (1) reliance upon existing curated rRNA databases to align sequences for taxonomic assignment and (2) lack of specificity in the resultant taxonomic categories. The first limitation signifies that only existing well-characterized species can be identified in the samples, with truly novel species falling into a generic unknown category. Pipelines such as QIIME provide a “*de novo*” strategy, which clusters sequence reads together without requiring a reference database, yet additional annotation is required to meaningfully interpret the results. The preferred approach is an “open reference” strategy that first aligns sequence reads to the reference database, and then uses clustering for any remaining reads. The latter limitation manifests as taxonomic categories typically at the family or genus level, and only rarely can specific species be identified. While this may be sufficient to give a general characterization of the microbial community, it offers little insight into particular strains or isolates of interest in clinical samples.

Despite its limitations, marker gene profiling is popular partly because it is inexpensive, thus allowing large data collection projects to be performed. The open challenge is how to use these large datasets to extract meaningful patterns and predictive models about microbial communities. For example, PICRUSt is a tool that predicts the functional capability of the microbiota by inferring the metagenome from the marker gene data [57]. Standard techniques for analyzing the functional composition of metagenomes including pathway enrichment and metabolic reconstruction can then be applied [58,59]. More fruitful approaches might utilize well-established machine learning algorithms to predict phenotypes, perform classification, and extract discriminative features [60-62]. Deconstructing the microbe-microbe interactions and internal community structure is challenging using only taxonomic abundances, but with perturbation data or time-series data then the various network inference algorithms used on gene expression data can be applied to the microbiome [63-66]. These can provide correlation networks [67,68], while methods with an underlying mechanistic model can provide predictive dynamics [49,69].

### 2.2. Metagenomics

Metagenomics requires substantial sequencing to be performed in order to obtain adequate depth and coverage of the microbiome, in contrast to marker gene profiling which only requires a few thousand sequence reads per sample. The presumption is the microbiome consists of numerous uncultivable species, so the sequence reads must be de novo assembled before being aligned to existing databases to catalog species and gene content. In theory with enough sequencing, whole genomes of novel species can be assembled [70-72]; however practically, de novo assembly tends to produces numerous short contigs (contiguous sequences). Producing high-quality drafts of microbial genomes will likely require borrowing techniques from eukaryotic genome assembly with longer read lengths and read-pair scaffolding [73,74], or possibly by utilizing single-cell sequencing technology [75,76]. However with the intense focus on the human microbiome, the number of fully assembled bacteria genomes is rapidly increasing, so the day may soon approach where the assembly step can be skipped in place of direct alignment to genome databases, and de novo assembly is reserved for the more diverse soil and marine ecologies. Alternatively, assembly-free methods could provide informative analysis with much less computational requirements [77,78].

Characterizing the gene content and associated functional composition of the microbiome is the initial descriptive analysis for metagenomic data, and the standard approach for determining gene content is to run BLAST, or similar alignment tools, on either the raw or assembled sequences against a reference database to find regions of local similarity (and presumed function) between sequences. For example, the MG-RAST pipeline uses the M5nr database, which is an aggregated set of non-redundant protein sequences from multiple sources [34,79]. Taxonomic categorization can also be performed by picking out just the marker gene sequences and is supported by the QIIME pipeline. Functional composition is generated by collating the functional categories assigned to the gene content from a set of functional databases such as KEGG Orthology [80] or SEED subsystems [31]. Specialized statistics tests can then be performed to compare the functional composition between experimental groups [81], and pathway analysis provides higher-level aggregate insights [58,82]. Comparative functional analysis between metagenomic samples is complicated by the fact that many bacterial genes have poor or unknown functional annotation and gene content of a sample is a mixture of multiple species [83]. Genes with metabolic functions tend to be well annotated, allowing the construction of mechanistic metabolic models [59,84,85], while gene regulatory and signaling networks are poorly characterized except for a small number of pathways in model organisms. As a mixture of species with unknown internal interactions, attempts at computational modeling of the microbial community require careful consideration of what constitutes a “community”, and the appropriate mapping of the functional composition with that community [26,86]. Furthermore, DNA measurement does not differentiate between alive or dead microbes and whether those functions are active.

Despite the difficulties, the broad unbiased approach of metagenomics is continuing to expand our catalog of known genes. The challenge of discovering the functions for many of these genes will likely require the development of high-throughput functional screens that can be applied to microbial communities [87]. Likewise, the largely unexplored world of bacterial phages and viruses in the intestine suggests another layer of complexity that will require metagenomics to adequately explore [88-90]. The primary challenge for metagenomics is determining the mechanistic underpinnings for changes in the microbiome, and deciding which changes are causal drivers with functional consequence *versus* less significant side effects. Integrating the metagenomics data with predictive computational models, such as is currently being done with metabolic modeling, provides one such route [91]. Including signaling pathways for virulence factors, reception and response to host and microbial factors and spatial organization of microbial communities will be key capabilities required for understanding the microbiome’s effect on human health. Another route is using dynamical system formulations of the microbial community and then inferring parameters from the experimental data [92], such as described above with marker gene profiling studies [49,69], but to date these techniques have not be applied to metagenomics data.

### 2.3. Metatranscriptomics

Metatranscriptomics requires RNA to be extracted from the samples, and unlike DNA techniques the experimental protocols are still being actively investigated to achieve both quality sequence and high yields. One of the experimental challenges for metatranscriptomics is the extraction of sufficient bacteria RNA to perform high-throughput sequencing without performing amplification. In combined host-microbiota *in vivo* experiments, even though the number of bacterial cells greatly outnumbers the host cells, they have much less total biomass, and host RNA dominates. Furthermore, the ribosomal RNA needs to be depleted from the bacterial RNA otherwise it will overpower the regulatory and signaling genes of primary interest. As a relatively new technique, there are numerous computational challenges to be addressed for metatranscriptomic data. The first is mapping of individual sequence reads to the appropriate genes. If only a functional characterization is required, then the techniques used to analyze metagenomic data can be used, as available with MG-RAST. Most desirable is to calculate gene expression values for the sequence reads and have them assigned to specific bacteria species. One approach is to collect a set of representative bacteria genomes and use fast alignment tools such as bowtie [93] or SSAHA [94] to align the sequences [43,95,96]. However, this only provides information for those species with a known genome, and furthermore will not work for clinical samples and bacterial isolates that have mutated significantly from laboratory strains. For example, our experience with a microbiome extracted from an intensive care unit patient [97] containing multi-drug resistant pathogens, *Enterococcus faecalis*, *Klebsiella oxytoca* and *Serratia marcescens*, found that only 1% of the sequence reads could be aligned to the reference genomes in NCBI using bowtie. Sequence alignment using BLAST of some randomly selected reads showed 90%–95% homology, which are more mismatches than efficiently allowed by these tools. Some studies use only BLAST [43,98], but this requires substantial computing capability to align millions of sequence reads. Current pipelines use a variant of this basic approach [99,100].

Bacteria share many common genes, especially those with metabolic function, and BLAST-style approaches produce numerous matches that require some heuristic post-processing analysis to assign the sequence read to a species. An alternative approach is to de novo assembly the sequence reads into transcripts, which can then be aligned against the transcripts to produce gene expression counts. The longer assembled transcripts will provide a more unique BLAST match for designating the species, as well as fewer sequences to process. Even this approach has its difficulties though as it relies upon the assembly quality. The sequencing depth may not sufficient to provide coverage across the whole transcript, possibly resulting in multiple assembled transcripts that partially cover the same gene or contain chimeric sequence. Furthermore, multiple bacterial genes are typically transcribed as a single unit, called an operon, so each assembled transcript can contain multiple genes that need to be individually parsed. None of the approaches currently published are optimal, as the ambiguity in the assignment of species-gene pair for each sequence read still needs to be solved. Functional analysis can be performed, but tools that provide comparative metatranscriptomic analysis, which highlights species transcriptional differences, is lacking. There are currently no published attempts to integrate metatranscriptomic data with mechanistic computational models. Regardless, metatranscriptomics holds great potential to elucidate the dynamics of the microbiota, and techniques to capture both host and microbiota transcriptomes will provide a broad snapshot of the host-microbiota interactome [99,101].

### 2.4. Computational Modeling and Simulation

Computational modeling and simulation of the microbiota has primarily focused on metabolic modeling due to the availability of genome-scale metabolic models for some bacteria [44,47], as well as software tools for automatically generating models from metagenomic data [45]. These approaches do not yet attempt to model the full complexity of the microbial community, instead the system is simplified using various abstractions. Models may focus on a single organism, a small community of interacting organisms or a supra-organism whereby all of the metabolic genes are aggregated together without regard to species [86]. The metabolic model is translated into a linear optimization problem where metabolic reactions become equality constraints for the reactants and products in the reaction. Inequality constraints are added to represent bounds on the system, such as the availability and rate that metabolites can be taken up from the environment, and an objective function is defined for the desired phenotype. Commonly a growth phenotype is desired, so an objective function that maximizes biomass is used. When the optimization problem is solved, it provides fluxes (or flows) on each reaction, the rate at which the metabolites are consumed or produced. This method is commonly called flux balance analysis (FBA) [46]. One critique of FBA metabolic models is the underlying assumption that the system is in steady state, which is likely false for *in vivo* systems, and the model does not take into account the availability and activity of the enzymes which catalyze those metabolic reactions. Also, FBA models utilize and provide metabolic rates, as opposed to metabolite concentrations, which are most often measured in experimental systems. As such, comparing FBA model results to experimental metabolomics data is challenging. However, the success of FBA models at predicting experimental phenotypes has led to new method development that addresses these criticisms, including incorporation of dynamics [102,103], integration with transcriptional regulation and signaling [103-107], and integration with “omics” data sets [108-110]. An interesting twist on these models is reverse ecology, which infers the set of compounds that the microbial community extracts from the environment [111].

Beyond metabolic models, dynamic modeling of the intestinal microbiota is still in its infancy [112]. Dynamic modeling has a long tradition in ecology where methods such as agent-based modeling were developed to represent heterogeneous agents interacting with each other in a heterogeneous environment [113], and these ecological methods are slowly being applied to the human microbiome. Agent-based modeling is highly applicable to microbial communities, however getting appropriate and sufficient experimental data to calibrate and validate the model predictions is still a formidable hurdle. Simplified population models such as the generalized Lotka-Volterra equations have been successfully used with marker gene profile data, and analysis of generated taxonomic interaction networks can provide insights about community stability or dysbiosis [49,69]. Another technique is to model bacterial as functional groups and to investigate the interactions of those groups. This approach was used to study antibiotic-mediated switch behavior with bacteria classified into functional groups from metagenomic data [48]. Additional multi-species modeling approaches are applicable to the intestinal microbiota including biofilm formation [114,115], cooperation and competition [116], and other ecological processes [117].

When considering individual bacterial species, there is a large body of dynamical modeling research that investigates specific molecular signaling and gene regulatory systems, behaviors and phenotypes. Such systems include cell-cell communication, quorum-sensing that provides population-level communication, motility on surfaces and in media, growth and colony pattern formation, environmental interactions through chemotaxis and haptotaxis, virulence factors related to infection and disease, genetic mutability, antibiotic resistance and numerous others. The major challenge in the scaling of these methods from individual pathways or modules to actual microbial behavior is the task of taking many small specific individual models and integrating them together to produce useful system-level models of bacteria. One route is to produce genome-scale models similar to metabolic models that have a high degree of fidelity in their component description, while using scalable numerical methods to examine the system dynamics [118]. An alternative route is to produce logical conceptual models that abstractly implement the low-level physical details of the biology while qualitatively representing our biological knowledge and hypotheses [119]. A combination of the two approaches will be the likely strategy in the immediate future. Regardless of the approach, extracting useful predictions and insights from increasingly complicated models will be a continuing obstacle.

## 3. Intestinal Host-Microbiota Interactome Studies

Computational modeling of the host-microbiota interactome is still in its infancy. The bioinformatics techniques to study the microbiota described in the previous section are helping to provide an increasing descriptive analysis about the microbial community, with the intestinal tract being one of most studied. On the other side of the interactome is the host immune system. There is a long history of immune system modeling. While this body of work is too large to adequately describe in this review, in the following sections we point out some main categories of interest. Consequently, we will highlight studies of intestinal inflammation where interactions with the microbiome can initiate, perpetuate or even disrupt the inflammatory response.

There are some significant methodological challenges that need to be addressed before causal mechanisms of the host-microbiota interactome can be effectively evaluated with computational modeling. Foremost is determining appropriate levels of representation for both the host and the microbiota, *i.e.*, what computational and mathematical constructs to use to represent the biological entities and processes. The host immune system manifests at the molecular, cellular, tissue, organ, whole organism and population scales of organization, and it is still an open question about how to define the interactions between these different scales as they can contain important inflection points of behavior [119-121]. The set of methods used in existing multiscale models would suggest that no single method would be sufficient for all purposes, as there are advantages and disadvantages with each. Meanwhile, microbes manifest at the molecular, cellular and population scales, and the quantity and diversity of individual bacteria presents computational challenges to specify the intra-community interactions in sufficient detail to establish dynamical changes in microbial community structure. The intestinal host-microbiota also exhibits spatial heterogeneity, with host cell responsiveness and microbial composition changing throughout the length of the intestinal tract. Moreover, the interactome acts through multiple routes including metabolite exchange and competition, signaling pathways mediated by extracellular molecules, physical interactions between host and bacteria cells, and the extracellular milieu which may be abundant with viruses, bacteriophages and mechanisms for horizontal gene transfer. Computational modeling is a knowledge-driven task, yet there are still large gaps in our biological knowledge about the interactome that can divert the modeling process. A combined data-driven and modeling framework will likely be the most effective approach to fill those gaps, with suggestive correlations from statistical analysis being evaluated for plausibility in mechanistic dynamical models.

### 3.1. Computational Modeling of Host Immune System

The host immune system is split between innate immunity and adaptive immunity. The innate immune system is the initial defense against infection and operates in a non-specific manner, while adaptive immunity invokes specialized responses to specific pathogens that are acquired during the lifetime of the host and maintained in immunological memory. Computational modeling of the immune system has historically concentrated either on specific host-pathogen interactions or on inflammation. Host-pathogen specific models include research on the diseases and pathogens with broad global health implications such as HIV [122-125], malaria [126-128], tuberculosis [129-131] and influenza [132,133]. These models primarily focus on the adaptive immune system and cover a wide range of topics related to the epidemiology of the diseases including the molecular biology interactions between host and pathogen pathways and molecules, disease progression and transmission both in the host and associated vectors (e.g., mosquito for malaria), evolutionary and selection forces on pathogen genetics, and drug discovery for vaccines and treatments. These models have helped to elucidate the dynamics associated with many of these processes, however no models have yet considered the microbiota, and biological studies of the microbiota’s relationship to these diseases are only beginning.

Computational studies of inflammation can be divided between acute and chronic inflammation, which both focus on, but consider different aspects of, the innate immune system [134-141]. Inflammation is a response by the immune system to injury or infection and involves three basic steps: (1) sensing of damage or threat; (2) containment and clearance of the threat; and (3) repair of the damaged tissue. Acute inflammation is the initial immune response to a threat, and it is a self-regulating system that, through a set of positive and negative feedbacks, will first upregulate immune processes and then downregulate them once the threat is removed [142-144]. Chronic inflammation is characterized by a disorder in the self-regulating inflammatory response system such that a persistent low-level inflammation continually damages and repairs host cells in localized tissue. For example, the disorder could be due to insufficient negative feedback that prevents the inflammatory response from completely turning off, or it could be excessive positive feedback that chronically re-starts the inflammatory response. Chronic inflammation can be due to genetic susceptibility in the host, the inability of the immune system to completely clear a bacterial infection or a combination of both. Early computational models emphasized acute inflammation, specifically in relation to trauma and sepsis, to better understand the self-regulating dynamics of the system for prevention and treatment of systemic inflammatory response syndrome (SIRS), a severe disruption of the innate immune system that can lead to organ dysfunction, failure and potentially death. While bacteria is known to cause or propagate the inflammatory response, these models generally define bacteria as an abstract perturbation to the host immune system, but there is increasing recognition that the interplay between host and microbial dynamics needs to be considered. Recently there has been greater interest in chronic inflammatory diseases such as necrotizing enterocolitis, Crohn’s disease and inflammatory bowel disease, and the role the microbiota can play on perpetuating or alleviating disease.

Computational models have used a variety of mathematical methods such as ordinary (ODE) and partial (PDE) differential equations [139,141,145], Boolean networks [146] and agent-based models (ABM) [139,144] to represent the host immune system. Furthermore, these models have to consider the inherent multi-scale nature of the immune system that include intracellular signaling networks, cell level behaviors and organ function. Though some models focus on just a single scale, which tends to be signaling and regulatory networks, other models incorporate multiple scales of organization and the interactions between scales to better represent the complexity of the immune system. The variety of applicable methods means that researchers can pick an appropriate level of abstraction based upon the types of available knowledge and data, and models can produce a spectrum of outcomes from quantitative results to qualitative thought experiments across the multiple scales [136,147,148].

### 3.2. Inflammatory Diseases and the Intestinal Host-Microbiota Interactome

The intestinal tract is subject to both acute and chronic inflammatory conditions. Acute intestinal inflammation can be part of a systemic response in sepsis and trauma, or due to intestinal surgery or injury, however current studies focus on individual opportunistic pathogens *vs.* the whole microbiota. Meanwhile, the microbiota and environmental factors that influence the microbiota have been shown to have a strong link with chronic intestinal inflammatory diseases [10,19]. The main challenge is how to translate the correlative descriptive studies about the changes in the microbiota and host immunity into causal mechanisms [149].

One step in that direction are attempts to overlay a generic dynamic model for the microbial community onto time-course data then analyze the resultant dynamical system for insights [48,49,69]. Stein *et al.* took this approach to hypothesize the colonization mechanism for *C. difficile* infection based upon the effect of antibiotics on the microbial community [49]. Marino *et al.* analyzed colonization of germ-free mice with the cecal contents of conventionally raised mice and suggested that few microbial interactions are mutualistic, while most are neutral Parasitic and competitive interactions dominated within specific phyla like Bacteroidetes and Firmicutes [69]. Both of these studies used the generalized Lotka-Volterra equations to define the interactions between microbial species in the community. Bucci *et al.* used a model derived from statistical physics to show that a two-group community composed of antibiotic-sensitive and antibiotic-resistant bacteria can exhibit multistability and hysteresis whereby the community can be dominated by either group [48]. Furthermore, it is an open question about how evolution formed stable microbial communities in the first place [150], especially given evidence that mutualism of the host-microbiota interactome is fragile whereby fast growing microbes can outcompete host beneficial ones [151]. Computational modeling is well suited to provide insights into the importance of interaction mechanisms and to evaluate hypotheses about these processes. Some recent models have considered interactions such as host genome evolution [152], economic market strategies [153], and the role of spatial structure [154,155]. There is a large body of theoretical ecology modeling research about food webs, biodiversity and community structure that can inform future microbial community modeling.

Spatial heterogeneity of the gut microbiota is well recognized, yet there are currently no computational models that consider the spatial host-microbiota interactome. However, there are models that consider the spatial architecture of the intestine in relation to disease and development that also include pathogen interaction. Three such models include a gastric mucosal immune response to *Helicobacter pylori* infection [156], *Pseudomonas aeruginosa* virulence activation in the pathogenesis of gut-derived sepsis [157] and dysentery resulting from *Brachyispira hyodysenteriae* infection [140]. Necrotizing enterocolitis is another intestinal disease that has received modeling attention [145,158,159]. The first paper develops a hybrid ODE and ABM model, with the ODE representing the signaling networks in and between four spatial compartments (lumen, epithelium, gastric lamina propria, gastric lymph nodes) while the ABM represents immune cell populations categorized by immunological states through the progression of *H. pylori* infection. The model depicts the migration of *H. pylori* from the mucus layer of the gastric lumen to invasion of the gastric lamina propria with spatiotemporal interactions between immune cells and spatial compartments. One of the challenges they identified was the lack of developed strategies for parameter estimation for ABMs in comparison to numerous methods available for ODEs, and thus because of the stochastic nature of ABMs, they needed to perform trial and error simulations to refine the parameters values. The high level of detail makes the model computationally expensive; the authors needed to use a high-performance computing cluster with 912 processor cores to run simulations. The *Brachyispira hyodysenteriae* infection model uses the same Enteric Immunity Simulator (ENISI) modeling environment as the previous model, and it describes in more detail the graphical discrete dynamical system that represents the spatial interaction between immune cell and bacteria agents. This method abstracts the spatial representation into compartments, which can be subdivided into sublocations. An agent’s location implicitly defines the set of other potentially interacting agents. This abstraction allows the full set of interactions to be computed, and the dynamical graph can be partitioned and executed in a parallel discrete-event simulation. Scaling to a larger number of agents is a difficult challenge for ABMs, and ENISI provides a novel approach for accomplishing large-scale simulations. The *Pseudomonas aeruginosa* virulence activation model developed by our group is an ABM with multiple virulence signaling pathways represented within *P. aeruginosa* as well as an abstract representation of the commensal microbiota. The commensal microbiota is represented collectively as generic microbial species without genetic background or detailed molecular mechanisms, however they compete with *P. aeruginosa* for nutrient resources within the intestinal lumen. Commensal and *P. aeruginosa* populations are limited by a finite carrying capacity per volume of the mucus layer, and *P. aeruginosa* activation of its virulence pathways can secrete chemicals that target the elimination of commensal microbiota. The model also represents gut epithelial cells as agents, and it consists of four spatial data layers for the intestinal lumen, mucous layer, epithelial layer and systemic circulation. This model takes a bacteriocentric viewpoint *vs.* the typical immunocentric perspective of other models by including detailed virulence pathways in *P. aeruginosa* while using simple behavioral rules for the host immune cells. This approach allowed for the exploration of hypotheses based upon current knowledge of bacterial virulence activation even though the model does not accurately capture all of the host dynamics. The authors note that adding in sufficient host immune system detail to obtain complete host dynamics would have made the model computationally intractable (or at least require a large compute cluster as with the other models), which would have defeated the purpose to engage in expedient “thought experiments” about plausible lines of investigation. As is, the model can be run on a single computer. This demonstrates the ongoing need to consider appropriate levels of representation when developing host-microbiota interactome models. The open challenge for the modeling community is the question of whether models are going to inherently become more detailed over time, and we just need to accept the increased computational costs, or whether we can develop methods that allow models at different abstract levels to be composed together. One approach is to utilize a more generic representation that includes semantic content such as biomedical ontologies for model specifications, and thus models can be composed and manipulated at the semantic level [160-162].

**Figure 2.**
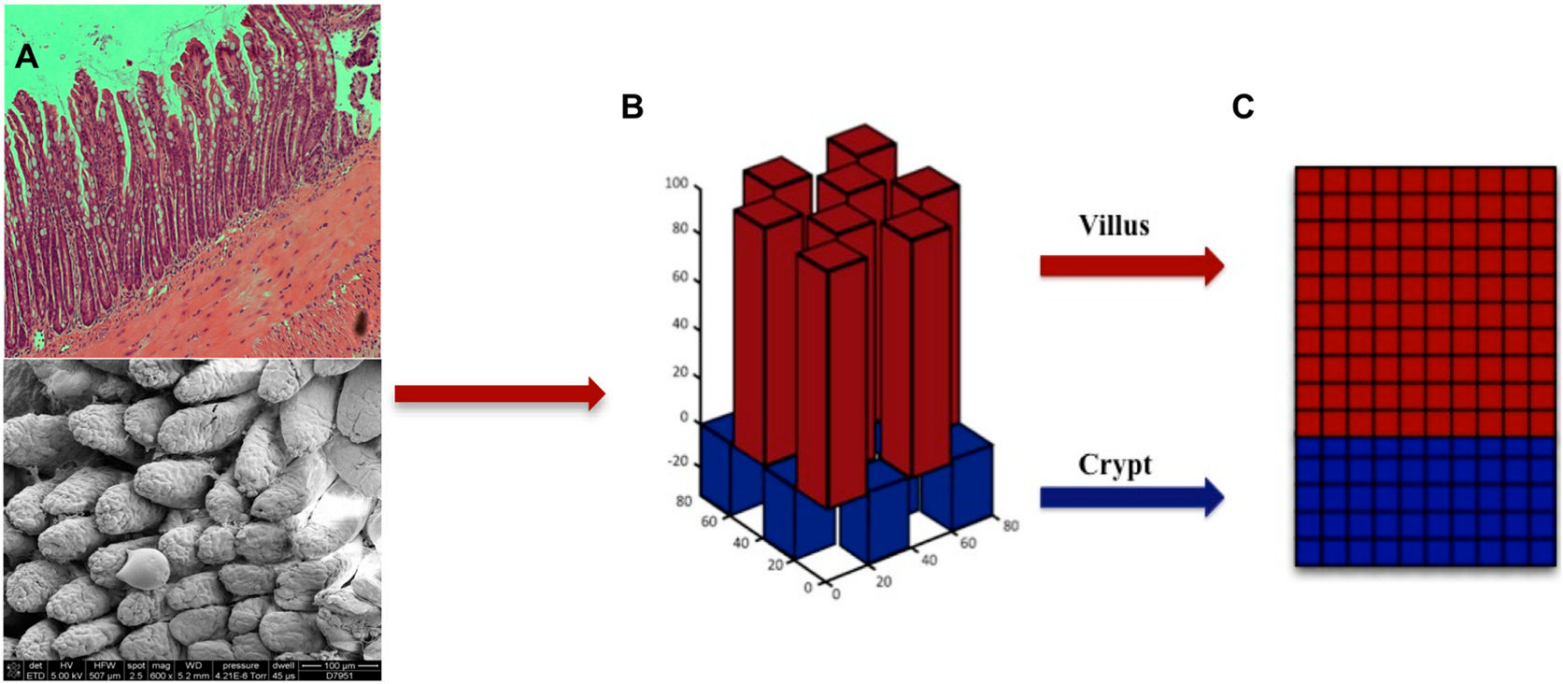
Crypt-villus architecture for Spatially Explicit General-purpose Model of Enteric Tissue (SEGMEnT) model. Panel (**A**) shows a histology cross section of ileal tissue (top) and scanning electron microscopy of the mucosal surface of ileum (bottom). Panel (**B**) is the topology used by SEGMEnT where crypts and villi are represented with a matrix of rectangular prisms. Each crypt and villus is mapped onto a two-dimensional grid (Panel (**C**)), where signaling interactions, morphogen diffusion and cellular actions take place. The topology can be replicated and extended to represent any size piece of tissue.

While the microbial community has spatial structure, that community can also alter the host epithelial intestinal architecture through the perpetuation of a chronic inflammatory response. Our group has published a model that considers this interplay of the host inflammatory response with the morphogenetic pathways that control spatial patterning and tissue architecture [163]. The Spatially Explicit General-purpose Model of Enteric Tissue (SEGMEnT) reproduces the epithelial crypt-villus architecture under health and disease conditions such as colonic metaplasia, which is characterized by a shift to a colonic tissue phenotype with increased crypt depth and shortened villi. SEGMEnT uses a 3D spatial representation for crypt-villus architecture (Figure 2), and this is in contrast to the spatial models described above which use compartments or an abstract 2D interaction grid. This more sophisticated spatial representation is required to accurately depict the morphogenetic changes to the crypt-villus architecture so that they can be correlated with experimental histology images. It is not a complete 3D representation however as cells are not represented within the interior of the villi or the spaces between the crypts, instead a 2D grid is mapped onto the 3D surface (Figure 2C). The 2D grid can change in height over time as cells are either lost or gained, and morphogen gradients define the crypt-villus boundary and may also shift as the morphogenetic functions are altered by host inflammatory input. Currently, the microbial interaction is represented as stimulatory input into the host inflammation pathway, and the microbiota is assumed to be in dysbiosis such that it maintains that input and generates a low-level chronic inflammatory response. However, in the future, SEGMEnT provides a framework for placing the microbial community in a spatial context from the intestinal epithelium to the lumen, and thus allows heterogeneous interactions to be modeled between microbes and host.

**Figure 3.**
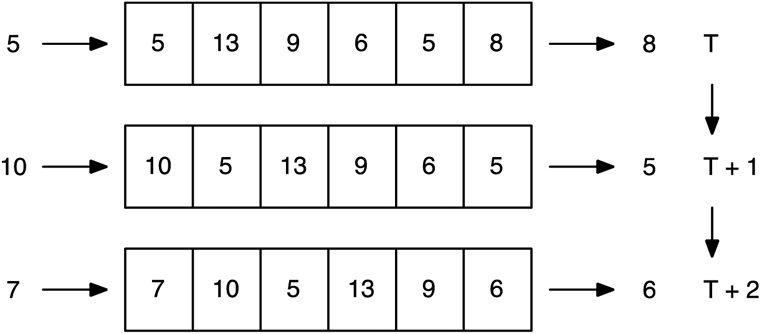
Queue data structure for time delay in a discrete simulation. Values that are produced, indicated on the left, are placed at the end of the queue structure. At each time step, values are shifted in the queue and the value at the front of the queue is removed. Progression of values is shown for two time steps, and the queue length represents a time delay of five time steps.

An uncommon feature that was implemented in SEGMEnT is time delays in the transcriptional machinery, translational machinery and transportation of the gene products for signaling networks. Standard continuous models (ODEs and PDEs) rely upon the reaction rate parameter values to characterize “slow” or “fast” interaction, which is physically accurate if all the detailed steps of an interaction are modeled, but oftentimes interactions are abstracted with many of the intermediate steps removed. In this case, the instantaneous nature of the continuous models is an approximation to the time delay from when a signal is received until the corresponding output is actualized. However, time delays can be incorporated in continuous models by defining functions that refer to the values of those functions at previous times; these are called delay differential equations (DDEs). While DDEs have been used in biological modeling [164-166], especially in models whereby such delays seem to be critical to behavior, their use is infrequent. Discrete simulations such as ABMs have an elegant method for implementing time delays by using a queue data structure to “hold” values for an appropriate amount of time steps until they are released. Figure 3 demonstrates how a time delay queue operates. Queues can be implemented efficiently using standard array structures, and they can readily support heterogeneous time delays for different products and even dynamically changing time delays. Within SEGMEnT, time delays were introduced for the transcriptional machinery based upon the average rate of transcription per nucleotide and the total nucleotide length for each gene. Time delays for the translational machinery was based upon the average rate of translation per amino acid and the total amino acid length for the protein, and transportation delays include moving products between the nucleus, the cytoplasm and the extracellular environment. Despite the ease of implementation, use of time delays in ABMs is also infrequent. For SEGMEnT, the objective for introducing time delays was to better correlate with the observed delays seen in experimental data, however the conclusion was that the time delays were insufficient to explain the experimental data. While such time delays could account for minutes of delay, it could not account for the multiple hours observed in experiments. It remains an open question about what degree of importance should be attached to the temporal calibration of models *vs.* qualitative reproduction of dynamic behavior. Including delays into a model that has feedback loops can introduce oscillations and other effects, yet it is also known that delays can benefit control [167]. This issue is relevant for host immune modeling because the whole system is geared around feedback control of sense and response, and the host-microbiota becomes another feedback layer that needs to be considered by the control circuitry. Accordingly, there is a growing collection of host immune system models that suggest time delays are an important factor to be considered [168-170].

Future work is needed to more explicitly define microbial communities. Specifically, the challenge is how to obtain sufficient functional characterization of a microbial community such that it can be used as an input into a mechanistic computational model, and correspondingly how model outcomes can be matched to experimental data. The host-microbiota interactome acts through multiple routes, yet it is experimentally difficult, if not cost prohibitive, to obtain the vertical depth of observation that includes metabolism, gene transcription, protein abundance, microbial composition, signal transduction, and environmental milieu. It is likely that we will never obtain complete breadth of observation except for highly controlled model organism systems. Instead, combining data-driven analysis with computational modeling will allow the limited experimental data to be incorporated as a way to both calibrate and constrain the exploration of causal mechanisms. Such studies are starting to be performed for inflammation [136]. Lagoa *et al.* utilized transcriptomic analysis of liver tissue to test hypotheses generated by a mathematical model of inflammation and global tissue damage [171]. Another study utilized principal component analysis of inflammatory regulators to suggest principal cytokine drivers of the inflammatory response, which was incorporated as putative hypotheses into an existing literature-based mathematical model [172]. By demonstrating that the modified model can recapitulate inflammatory and physiologic responses, the results provide plausible new knowledge about blood-lung inflammatory interactions. In both studies, the experimental data is not quantitatively calibrated with modeling variables, which is often not possible for complicated biological processes such as inflammation as those variables do not have a direct physical correlate. Instead, the first study used clustering and pathway analysis of the gene expression data to provide correlation with the modeling outcomes, while the latter study suggested potential interactions between biological components with the functional form of the interaction provided by the researchers. These studies illustrate that combining data-driven and mechanistic modeling is not a straightforward process of matching variables to data, instead metrics and higher-level analysis needs to be performed, an area of research that requires increasing attention as more of these studies are performed.

An exciting future prospect for computational modeling is the development of *in silico* clinical trials to test the efficacy of therapies and interventions [147,173,174]. Known human variation is incorporated into the computational model, and patient cohorts are created with a set of randomly generated models. Simulations are performed for a control group and an intervention group of patient cohorts, and model outcomes are assessed to determine the effect of the intervention. Clermont *et al.* applied this idea to an anti-TNF (tumor necrosis factor) therapy. They were able to identify a window of opportunity when the therapy was effective, but also characterized a population that could be harmed by the therapy [173]. An alternative utilization of *in silico* clinical trials, with applicability to personalized medicine, is to analyze the modeling outcomes to define the characteristics of patients that would most benefit from the therapy, and modify the patient selection process accordingly when performing a human clinical trial [175]. With regards to the host-microbiota interactome, the accumulation of microbiome data in sequence databases provides a rich description of the microbiome variation present in humans in healthy and diseases conditions. By explicitly incorporating this variation into host-microbiota models, the complexity of the microbiota can serve to constrain the interactome in regions of dynamic stability that characterize appropriate host and microbiota responses. However, an appropriate mathematical characterization of microbiome variation has yet to be developed. To fully realize such models also requires gathering host genetic variation and other data applicable to host immunity modeling.

## 4. Conclusions

The host-microbiota interactome is the next frontier in the nascent field of translational systems biology and bioinformatics. The large collection of host immunity computational models plus expanding microbiome databases provide fertile ground for combining knowledge-driven computational modeling with data-driven bioinformatics in the development of new methods and analyses. Methodological progress is required in order to significantly advance our knowledge of the host-microbiota interactome and its impact on human health. In this review, we have highlighted some of the main challenges and existing gaps with the hope that it will encourage research in new computational methods. For microbiome analysis, the primary challenge is extracting functional characterization of the microbiota. New methods are required to analyze metatranscriptomic data, and integration of multiple data types is needed to provide a more complete reconstruction of the microbial community. While there has been significant progress in the metabolic modeling of individual microbial species, these models need to be extended to encompass a community of interacting microbes and include signaling and regulatory networks. The disparate collection of knowledge about signaling and regulatory genes and pathways in microbial species needs to be combined into more comprehensive whole organism models. This is not purely an exercise of collecting together the parts list of physical components; instead multiple techniques for abstracting that knowledge into functional models should be devised to address the multi-scale nature of biology. This review has focused on the host-microbiota interactome in the human gut, with emphasis on the host immune system response, though many of these challenges apply to other epithelial surfaces and host organisms. Finally, existing host immunity computational models need to incorporate the microbiota and its interaction with the host. Accomplishing this integration requires appropriate representation of the microbiota so that it can be coupled with the host in a dynamic computational model. Furthermore, new metrics and analysis techniques are needed to correlate modeling outcomes with experimental data for calibration, validation and prediction. The development and utilization of comprehensive, multi-scale, validated computational models of the intestinal host-microbiota interactome could have profound relevance for clinical practice in the treatment and prevention of trauma, sepsis, surgical infections and chronic inflammatory diseases, as well as a better understanding for the role that diet, stress and other environmental factors influence our health.
